# RPP30, a transcriptional regulator, is a potential pathogenic factor in glioblastoma

**DOI:** 10.18632/aging.103596

**Published:** 2020-07-23

**Authors:** Guanzhang Li, You Zhai, Hanjie Liu, Zhiliang Wang, Ruoyu Huang, Haoyu Jiang, Yuemei Feng, Yuanhao Chang, Fan Wu, Fan Zeng, Tao Jiang, Wei Zhang

**Affiliations:** 1 Department of Molecular Neuropathology, Beijing Neurosurgical Institute, Capital Medical University, Beijing, China; 2 Department of Neurosurgery, Beijing Tiantan Hospital, Capital Medical University, Beijing, China; 3 Center of Brain Tumor, Beijing Institute for Brain Disorders, Beijing, China; 4 China National Clinical Research Center for Neurological Diseases, Beijing, China; 5 Chinese Glioma Genome Atlas Network (CGGA) and Asian Glioma Genome Atlas Network (AGGA)

**Keywords:** glioblastoma, RPP30, age, RNA modification, pathogenic factor

## Abstract

Background: Old age has been demonstrated to be a risk factor for GBM, but the underlying biological mechanism is still unclear. We designed this study intending to determine a mechanistic explanation for the link between age and pathogenesis in GBM.

Results: The expression of RPP30, an independent prognostic factor in GBM, was negatively correlated with age in both tumor and non-tumor brain samples. However, the post-transcriptional modifications carried out by RPP30 were different in primary GBM and non-tumor brain samples. RPP30 affected protein expression of cancer pathways by performing RNA modifications. Further, we found that RPP30 was related to drug metabolism pathways important in GBM. The decreased expression of RPP30 in older patients might be a pathogenic factor for GBM.

Conclusion: This study revealed the role of RPP30 in gliomagenesis and provided the theoretical foundation for targeted therapy.

Methods: In total, 616 primary GBM samples and 41 non-tumor brain samples were enrolled in this study. Transcriptome data and clinical information were obtained from the CGGA, TCGA, and GSE53890 databases. Gene Set Variation Analysis and Gene Ontology analyses were the primary analytical methods used in this study. All statistical analyses were performed using R.

## INTRODUCTION

Glioblastoma (GBM) is the most common primary intracranial malignancy in adults [1, 2]. It is highly lethal, with a median survival of only about 14.4 months [3]. An improved understanding of mechanistic causes of GBM may provide a foundation for the improvement of treatment outcomes [4]. Indeed, significant efforts in recent years have explored the molecular pathogenesis of GBM [5].

With the development of high-through sequencing technology, detailed analyses of genetic associations in the pathology of GBM have accelerated. In the past few years, many disease-associated genetic variations in GBM have been documented. Chromosome 7 amplification, chromosome 10 deletions, EGFR amplification, EGFR mutations (point and vIII mutations), and PTEN deletion are high-frequency mutations in primary GBM [6–8]. The pathogenic mechanism of these mutations in GBM has been extensively studied [9–11]. However, the association between age, a risk factor for many tumors, and glioma have been relatively understudied. Epidemiological studies suggest that nearly 80% of gliomas occur in middle-aged and elderly patients [12, 13]. Therefore, we speculated that gliomas occurred more frequently in elderly patients due to the accumulation of genetic mutations or possibly changes in transcriptomics.

Ribonuclease P protein subunit p30 (RPP30), a component of ribonuclease P (RNase P), generates mature tRNA molecules by cleaving the 5'-end. RNase P is also known as RNA polymerase III and is one of three major nuclear RNA polymerases in human cells. Studies have shown that in addition to the modification of tRNA, RNase P is involved in the regulation of gene transcription and cell cycle [14, 15]. Previous work has reported that RPP30 plays a role in tumorigenesis and malignant progression in breast, ovarian, and lung cancers [16–18]. Furthermore, some studies have studied RNase P as a potential therapy for several cancers [19, 20]. Therefore, we speculated that RPP30 may play an important role in glioma with potential as a novel therapeutic target.

In this article, we studied the relationship between age and gliomagenesis. First, age-related genes were screened in primary GBM and non-tumor brain samples. Functional enrichment analysis found that these genes were closely related to gene transcription. Compared with non-tumor brain samples, we found a loss of post-translational protein modification of RPP30 in GBM. In-depth studies have found that RPP30 expression affected the post-transcriptional modification of tumor pathway genes, which may be one of the causes of primary GBM. Finally, we found that RPP30 was closely associated with the clinical molecular pathological features and transcriptional modification of GBM. In conclusion, we found that RPP30, which is expressed less with age, maybe one of the pathogenic factors leading glioma, and RPP30-targeted therapy could potentially be used for clinical treatment of GBM patients.

## RESULTS

### Age-related genes were responsible for transcriptional regulation in primary GBM and non-tumor brain samples

**Figure 1:**
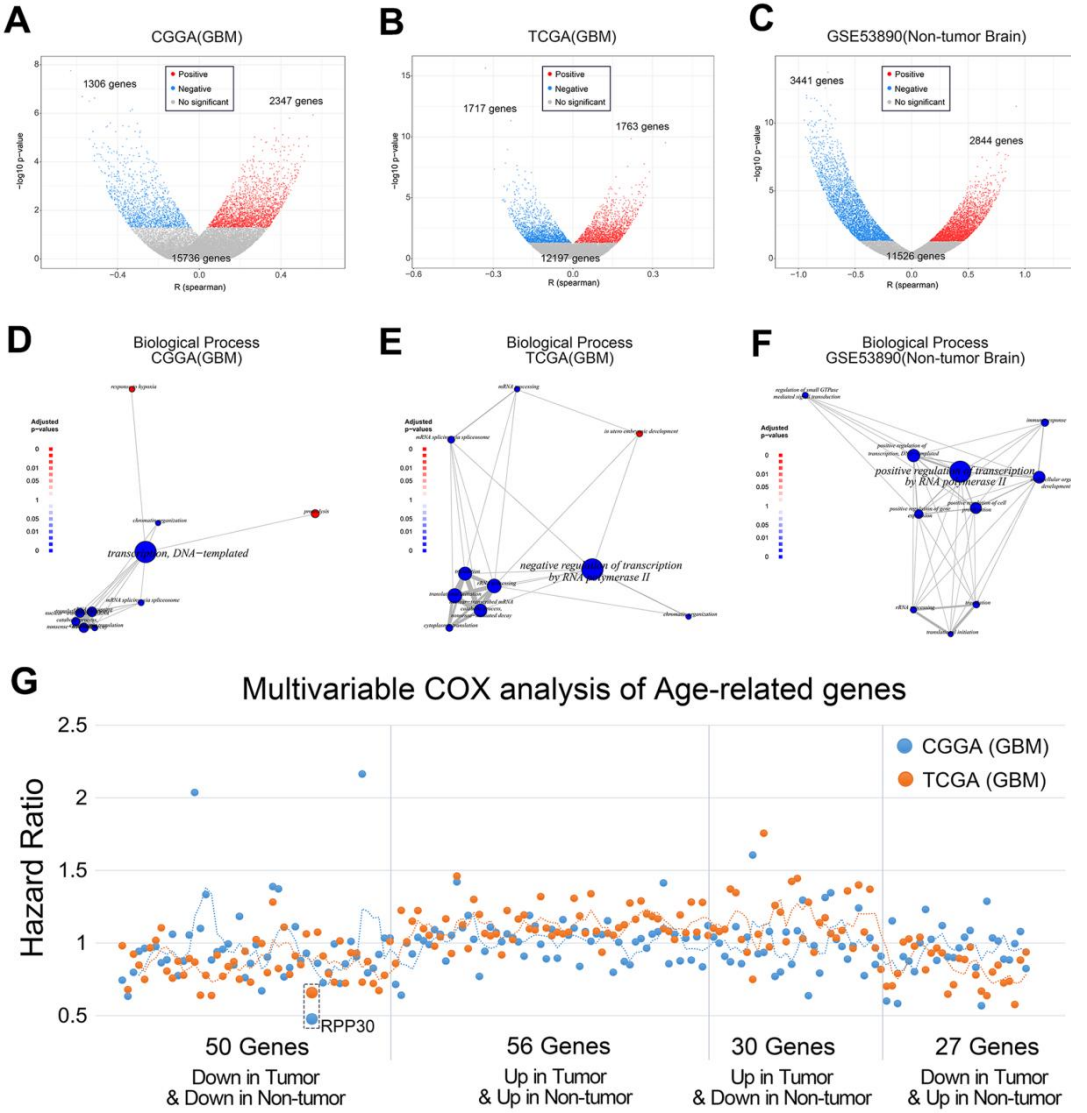
**Age-related genes are mainly enriched in transcriptional regulation.** (**A**, **B**) The correlation between gene expression and age of primary GBM in CGGA and TCGA databases. (**C**) The correlation between gene expression and age of non-tumor brain samples in GSE53890. The statistical significance between age and gene expression was assessed by Pearson correlation analysis. (**D**, **E**) Functional enrichment of age-related genes of primary GBM in CGGA and TCGA databases. (**F**) Functional enrichment of age-related genes of non-tumor brain samples in GSE53890. (**G**) Multivariable COX analysis of Age-related genes in primary GBM. Among the above genes, only RPP30 was an independent prognostic factor by multivariate COX analysis. Multivariate COX analysis of age-related genes was performed separately.

At first, we speculated that the accumulation of gene mutations with age was the main cause of gliomagenesis. However, our results suggested that the number of gene mutations does not accumulate with age in samples from the TCGA (Supplementary Figure 1A–1C) and CGGA (Supplementary Figure 1D–1F) databases. Next, we investigated age-related gene expression in primary GBM and non-tumor brain samples. We found that whereas gene expression was negatively correlated with age in non-tumor brain samples, gene expression in primary GBM was upregulated (Figure 1A–1C). Subsequently, we performed functional enrichment analysis on genes closely related to age in each database. As shown in Figure 1D–1F, age-related genes were primarily responsible for transcriptional regulation both in GBM samples and non-tumor brain samples.

### RPP30, which decreases with age, was an independent prognostic factor in primary GBM

To explore the prognostic significance of age-related genes in primary GBM patients, we performed the following analyses. First, age-related genes from primary GBM and non-tumor brain samples were divided into 4 groups: those whose expression was either up or down in both primary GBM and non-tumor brain samples (56 genes and 50 genes respectively), up in primary GBM but down in non-tumor brain samples (30 genes), or down in primary GBM but up in non-tumor brain samples (27 genes). Subsequently, we performed multivariate COX analysis on these 163 genes including IDH1 mutation status and chemoradiotherapy. Finally, we selected RPP30, an age-related and independent prognostic factor, as the only candidate gene for further study (Figure 1G).

### RPP30 behaved differential post-transcriptional modifications in primary GBM brain samples

**Figure 2:**
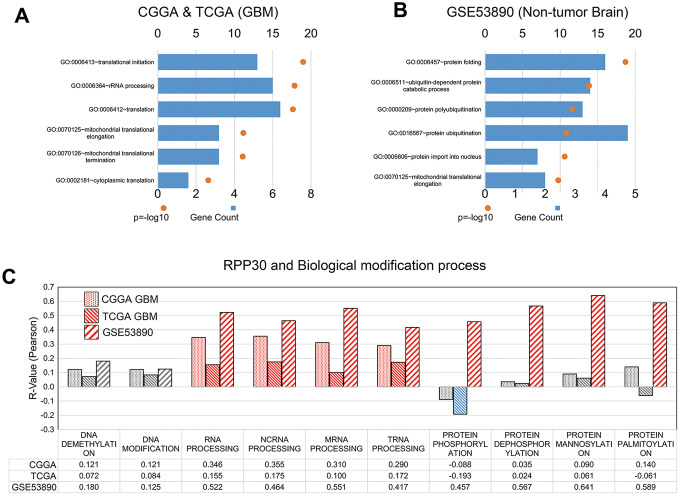
**RPP30 involved in the post-translational modification in GBM and non-tumor brain samples.** (**A**) Gene ontology (GO) analyses of RPP30 in GBM. Functional annotation of 500 genes most correlated to age in both CGGA and TCGA databases. (**B**) Gene ontology (GO) analyses of RPP30 in non-tumor brain samples. Functional annotation of 500 genes most correlated to age in GSE53890. (**C**) Correlation between RPP30 and GSVA scores of DNA, RNA, and Protein modification in CGGA, TCGA, and GSE53890. Red columns represented significant positive correlation. Blue columns represented significant negative correlation. Gray columns represent no significant correlation. The statistical significance was assessed by Pearson correlation analysis.

We conducted gene ontology (GO) term enrichment analyses to determine the function of RPP30 in primary GBM and non-tumor brain samples. We found that whereas RPP30 in primary GBM was primarily associated with translation-related functions, it was associated with protein ubiquitination and folding functions in non-tumor brain samples (Figure 2). This result suggested that RPP30 plays a role in different stages of post-transcriptional modifications in primary GBM and non-tumor brain samples. Further, we explored the specific modification functions of RPP30 at each stage of gene expression. Consistent with previous research findings, we found that RPP30 was involved in the modification of RNA, not DNA, in both tumor and non-tumor samples [14, 15, 18]. Surprisingly, we found that RPP30 was closely related to protein modification in non-tumor brain samples, but not in primary GBM samples in both the CGGA and TCGA databases (Figure 2C).

### RPP30 was correlated with tumor-associated signaling pathways

**Figure 3:**
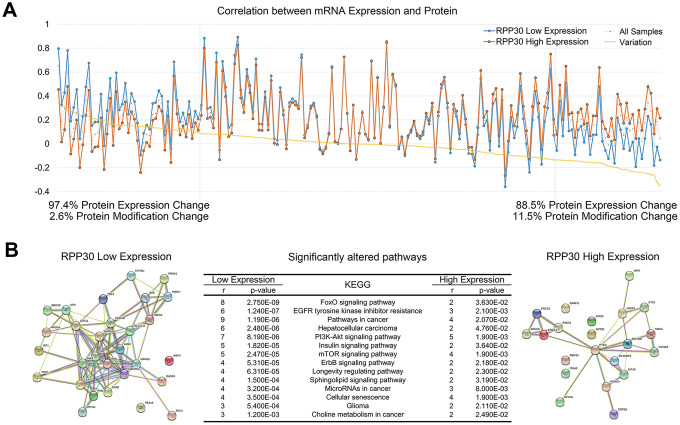
**Protein expression in cancer pathways was affected by post-transcriptional modification of RPP30.** (**A**) The correlation between protein expression and mRNA expression was affected by RPP30 expression in GBM. (**B**) Functional protein association network analysis of proteins regulated by post-transcriptional modification of RPP30 in STRING. Pathway enrichment results of proteins in RPP30 high and low groups were shown in the table.

Prior studies from our lab and others have confirmed that RPP30 is involved in the post-transcriptional modification in tumors [19, 21]. Therefore, we analyzed the relationship between RPP30 and RNA modification in primary GBM. We found that the correlations between mRNA and their corresponding proteins were different in RPP30-low and RPP30-high GBM samples (Figure 3A). However, this divergence was mainly (~90%) caused by a difference in protein expression rather than protein modification status. This result indicated that RPP30 influenced the translation of select proteins in an RNA-modification-dependent manner. Functional enrichment analysis of the proteins that receive post-transcriptional modifications from RPP30 revealed that those proteins were mainly involved in the activation of cancer signaling pathways (Figure 3B). The relevant cellular signaling pathways are summarized in Figure 3B. These results suggested that RPP30 may regulate tumor-associated signaling pathways by modifying the mRNA of key corresponding proteins.

### RPP30 was closely related to clinicopathological characteristics of primary GBM

**Figure 4:**
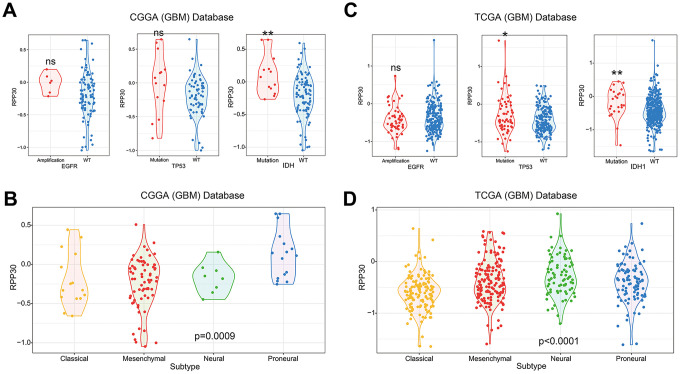
**The correlation between RPP30 and clinicopathological characteristics in primary GBM.** (**A**, **C**) RPP30 was enriched in TP53 mutation and IDH1 mutation GBM samples in CGGA and TCGA databases. The expression of RPP30 was independent of the amplification state of EGFR. The unpaired t-test was used in differential analysis. ns: no significant difference. *: p<0.05. **: p<0.01. (**B**, **D**) Expression pattern of RPP30 in four transcriptome subtypes of GBM. One Way ANOVA was used in differential analysis.

In light of the important functions of RPP30, we next explored its relationship with the clinicopathological characteristics of primary GBM. Our analysis of the CGGA and TCGA databases suggested that RPP30 was enriched in GBM samples with TP53 mutation and IDH1 Mutation, but was unrelated to EGFR amplification status in (Figure 4A, 4C). Transcriptional subtype was an important molecular pathological feature of GBM. Increased expression of RPP30 was detected in the subtype of neural and proneural subtypes and was associated with better prognosis (Figure 4B, 4D). However, there was no significant correlation between RPP30 and tumor purity or gene mutation numbers (Supplementary Figure 2). Further, the expression of RPP30 in primary GBM patients was not related to the sensitivity to postoperative radiotherapy and temozolomide (Supplementary Figure 3). These results revealed the close relationship between RPP30 and clinicopathological characteristics of primary GBM.

### RPP30 played a role in transcriptional regulation in primary GBM

**Figure 5:**
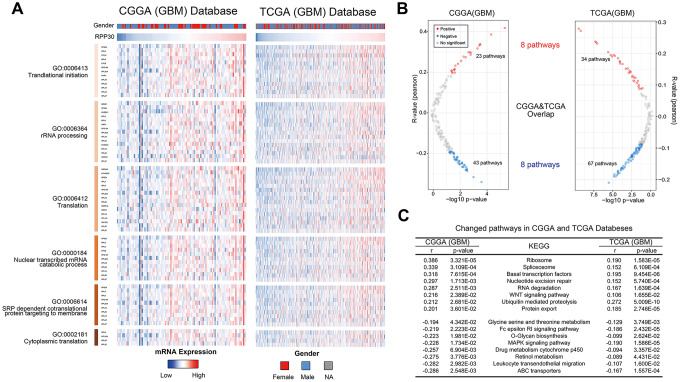
**Expression pattern and pathways associated with RPP30 expression in primary GBM of CGGA and TCGA databases.** (**A**) The heatmap showed the expression pattern of RPP30 and transcription-related genes in GBM. Transcription-related genes were obtained from the AmiGO 2 Web portals. Besides, there was no difference in RPP30 expression between different genders of GBM. (**B**) The scatter plot showed the pathways closely related to RPP30 in CGGA and TCGA databases. There were 8 pathways positively and 8 pathways negatively correlated with RPP30 expression in both CGGA and TCGA databases. (**C**) The correlation coefficient and p-value of the above 16 pathways with RPP30 were shown in the table. The statistical significance was assessed by Pearson correlation analysis.

To further illustrate the role of RPP30 in transcriptional regulation, we created heatmaps of RPP30 and transcription-related genes in primary GBM. We found that RPP30 and transcription-related genes shared the same expression pattern in CGGA and TCGA databases (Figure 5A). These genes and their corresponding correlation coefficients are shown in Supplementary Table 3. In addition, we also found that there was no differential expression of RPP30 in male and female patients. This result further suggested that RPP30 plays a role as a transcriptional regulator in primary GBM.

### RPP30 activated cancer and drug metabolism pathways

To further explore the biological functions of RPP30, we performed a correlation analysis between RPP30 and 186 KEGG pathways in primary GBM. We found 8 positively and 8 negatively correlated pathways associated with RPP30 in the CGGA and TCGA databases (Figure 5B). In addition to transcriptional modification, RPP30 was positively correlated with the WNT signaling pathway (pro-cancer) and negatively correlated with several drug metabolism pathways (cytochrome p450 and ABC transporters) (Figure 5C). Meanwhile, the expression level of RPP30 was significantly correlated with the expression of genes in the cancer-related pathways (Pathway in cancer, WNT Pathway, MAPK Pathway, and WNT Pathway) in both CGGA and TCGA databases (Supplementary Figure 4). These results suggested possible differential biochemical and functional outcomes triggered by RPP30 that may finally result in poorer prognosis in older patients.

### RPP30 affected cell proliferation and pathway activation *in vitro*

**Figure 6:**
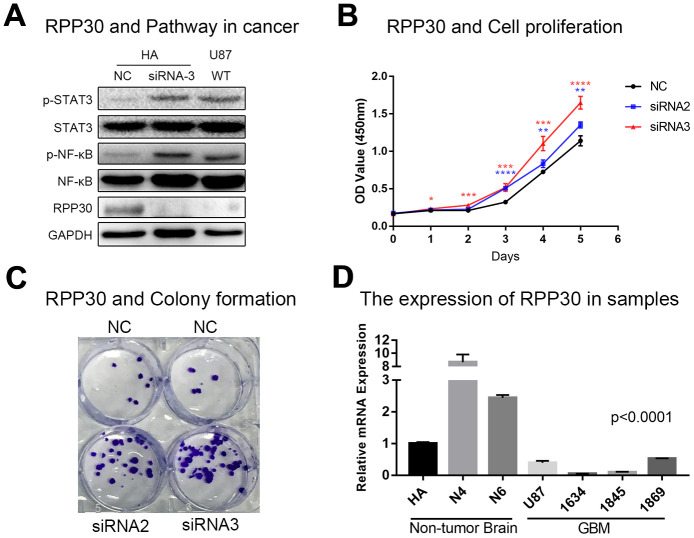
**RPP30 regulated protein activation and cell proliferation in vitro.** (**A**) Western blot showed knockdown of RPP30 led to increased expression of p-STAT3 and p-NF-κB in HA cells. (**B**, **C**) Cell proliferation ability increased significantly after knocking down RPP30 in HA cells. (**D**) RPP30 was lowly-expressed in GBM samples by qRT-PCR.

To verify the function of RPP30, we performed *in vitro* experiments. We found that knockdown of RPP30 mRNA expression in HA cells led to the activation of the STAT3 and NF-κB pathways (Figure 6A). Further functional experiments found that knocking down RPP30 increased the proliferation of HA cells (Figure 6B, 6C). In contrast, the activation of tumor-related pathways and proliferation ability of HA cells were impaired by the over-expressed RPP30 (Supplementary Figure 5). Further, we measured the relative expression of RPP30 in non-tumor and GBM samples via qRT-PCR. We found that RPP30 was lowly-expressed in GBM samples (Figure 6D). Together, these results indicate that RPP30 may contribute to the pathogenesis of GBM.

## DISCUSSION

Due to our limited understanding of the pathogenesis of GBM, current postoperative treatment is mainly untargeted adjuvant therapy, which is largely ineffective for most patients and has poor prognoses [1, 22]. However, the launch of the TCGA and CGGA projects have catapulted GBM research into a new era. In just the past decade, there have been thousands of high-throughput sequencing studies in GBM. Many genomic alterations in GBM have been identified, including EGFR amplification, EGFR mutations (point and vIII mutations), PTEN deletion, and others [23–26]. However, the primary causes of GBM progression remain unknown. Therefore, individualized targeted therapy is only able to benefit a small number of GBM patients. Therefore, the clinical community is in urgent need of an improved understanding of fundamental pathogenic processes that could serve as therapeutic targets. Advancing age is considered to be the pathogenic factor of many cancers [27, 28]. Previous studies have suggested that age-related epigenetic alterations, accumulated mutation, cell metabolic reprogramming, and changes to the tumor microenvironment play an important role in tumorigenesis [29–37]. This study was designed to explore the mechanistic relationship between age and the pathogenesis of primary GBM.

Unexpectedly, the number of gene mutations did not increase with age in primary GBM. Therefore, we studied the relationship between transcriptome and age. Analysis of transcriptome sequencing data of GBM and non-tumor brain samples revealed that transcription of 163 genes is closely related to age. Importantly, these genes were primarily found to be involved in gene transcription in both tumor and non-tumor brain samples. This suggests that differential epigenetic regulation of the transcriptome is closely related to age in tumor and non-tumor brain samples. Further survival analysis found that only one particular gene - RPP30 - was an independent prognostic factor for primary GBM. Previous studies reported that RPP30 played an important role in gene transcription, with a primary role in making post-translational modifications [38]. Our study found that RPP30 was mainly involved in the post-translational modification in both tumor and non-tumor samples. However, we also found that the specific post-transcriptional modifications made by RPP30 were different in tumor and non-tumor brain samples. Our data suggested that RPP30 was associated with RNA and protein modification in non-tumors but associated with RNA modification in GBM tissue. Indeed, this hypothesis was corroborated by the finding that RPP30 could regulate protein expression, rather than protein modification, by post-transcriptional modification in GBM. The protein regulation role of RPP30 may be an important potential pathogenic factor in GBM. Functional enrichment of RPP30-related proteins showed that these proteins were mainly enriched in cancer-related pathways. In addition, we found that the downregulation of RPP30 can increase phosphorylation/activation of cancer pathway-associated proteins *in vitro*. Whereas overexpression of RPP30 has the opposite biological functions. Since there is no anti-RPP30 antibody available for immunohistochemistry, we measured the relative expression of RPP30 in non-tumor and GBM brain samples via qRT-PCR. Our findings suggested that RPP30 was lowly expressed in GBM samples. These results suggest that RPP30 might act as a pathogenic factor in GBM by carrying out post-translational modifications of cancer pathway-related proteins. However, significant further research of how these findings may inform a therapeutic strategy is required before translation to the clinic.

Herein, we reported for the first time an analysis of the biological functions of RPP30 in glioma. We found a relationship between RPP30 and specific hotspot mutations and molecular subtypes in GBM. Further, we analyzed RPP30-related transcriptional regulatory genes and molecular pathways. Of interest, the RPP family has previously been studied as a therapeutic target in the treatment of tumors [19]. Our results from this work validate the possibility that RPP30 may be an important pathogenic pathway in the development of GBM. We found that RPP30 likely acts via post-transcriptional editing of select oncogenes. With follow-up developmental studies, we believe that RPP30-targeted therapy might benefit GBM patients.

## MATERIALS AND METHODS

### Sample and database

In the CGGA database, we have collected transcriptome data from 109 primary GBM samples, originally generated by the Agilent Whole Human Genome Array platform. Overall survival (OS) was calculated from the data diagnosed as the time of death or last follow-up. Written informed consents were obtained from the patients (or their families) for the CGGA project. We downloaded transcriptome microarray data from 507 samples of primary GBM from the official website of TCGA (https://cancergenome.nih.gov). Transcriptome microarray data from non-tumor brain samples were obtained from GSE53890.

### Cell culture

We obtained GBM cell line U87 and astrocytic cell line HA from the Institute of Biochemistry and Cell Biology, Chinese Academy of Science. U87 cells were cultured in DMEM (Gibco) supplemented with 10% fetal bovine serum (Gibco). HA cells were cultured in Astrocyte Medium (Gibco). All cell lines were cultured at 37 °C and 5% CO_2_.

### Screening of candidate genes

Age-related genes were screened out by *pearson* correlation analysis in primary GBM (from CGGA and TCGA databases) and non-tumor brain samples (from GSE53890). We found 163 genes to be significantly associated with age in both primary GBM and non-tumor brain samples and included those for further analysis (Supplementary Table 1). To explore the prognostic significance of 163 genes in primary GBM, we performed multivariate COX analysis in CGGA and TCGA databases. Finally, we selected RPP30, an age-related gene and prognostic factor of primary GBM, as the candidate gene of focus for the rest of our studies.

### Functional enrichment analysis

In this study, we used three functional enrichment methods. We performed functional enrichment of age-related genes by the *HTSanalyzeR* package in R. The p-value cutoff was 0.01, permutations were 100, and minGeneSetSize was 20. Gene ontology (GO) analyses of RPP30 in GBM and non-tumor brain samples were performed in DAVID (https://david.ncifcrf.gov/). The functional enrichment of RPP30-related proteins was performed in STRING (https://string-db.org/).

### Gene Set Variation Analysis (GSVA) analysis

GSVA analysis was performed using the *gsva* package in R. Analysis was performed using the default parameters. The Gene Ontology (GO) gene set was downloaded from AmiGO 2 Web portals (http://amigo.geneontology.org/amigo/landing). The correlation between genes and biological functions were analyzed by *pearson* analysis.

### Cell proliferation test and colony formation

We conducted a cell proliferation test using the Cell Counting Kit-8 Kit (Dojindo). For the colony formation assay, we plated dissociated single cells at a density of 1 cell/μl and counted the number of colonies that formed after 14 days.

### Quantitative Real-time PCR

Expression levels of mRNA were analyzed using the ABI 7500 Real-time PCR System. We calculated the relative mRNA expression levels of RPP30 using the 2^–ΔΔCt^ method. Transcript levels of the GAPDH gene were used for normalization. The primer sequences for various human genes used in this study are listed in Supplementary Table 2.

### Statistical analysis

Statistical analyses and figures were executed using R (https://www.r-project.org/, v3.5.0), SPSS software (IBM, v25.0), and Microsoft office 2016. We used SPSS for multivariable COX analysis and the results were depicted using Microsoft office 2016. Other statistical computations and figures were generated using R packages. A p-value less than 0.05 was considered statistically significant. All statistical tests were two-tailed.

### Ethics statement

This study was approved by Beijing Tiantan Hospital institutional review board (IRB). All patients provided written informed consent for the publication of all associated data in this study.

### Availability of data and materials

The sequencing data of the CGGA database has been published on the CGGA portal website (http://www.cgga.org.cn/). The sequencing data of the TCGA database could be downloaded from the official website of TCGA (https://cancergenome.nih.gov). Transcriptome microarray data from non-tumor brain samples were obtained from GSE53890 (https://www.ncbi.nlm.nih.gov/geo/query/acc.cgi?acc=GSE53890). All data analyzed during the study are available from the corresponding author on reasonable request.

## Supplementary Material

Supplementary Figures

Supplementary Table 1

Supplementary Tables 2 and 3
