# Supplementary material for: RPP30, a transcriptional regulator, is a potential pathogenic factor in glioblastoma
Source: Aging (Albany NY). 2020 Jul 23;12(16):16155–71. doi: 10.18632/aging.103596 (PMC7485703; doi:10.18632/aging.103596)
Supplement: Supplementary Figures [file aging-12-103596-s003..pdf]

SUPPLEMENTARY FIGURES

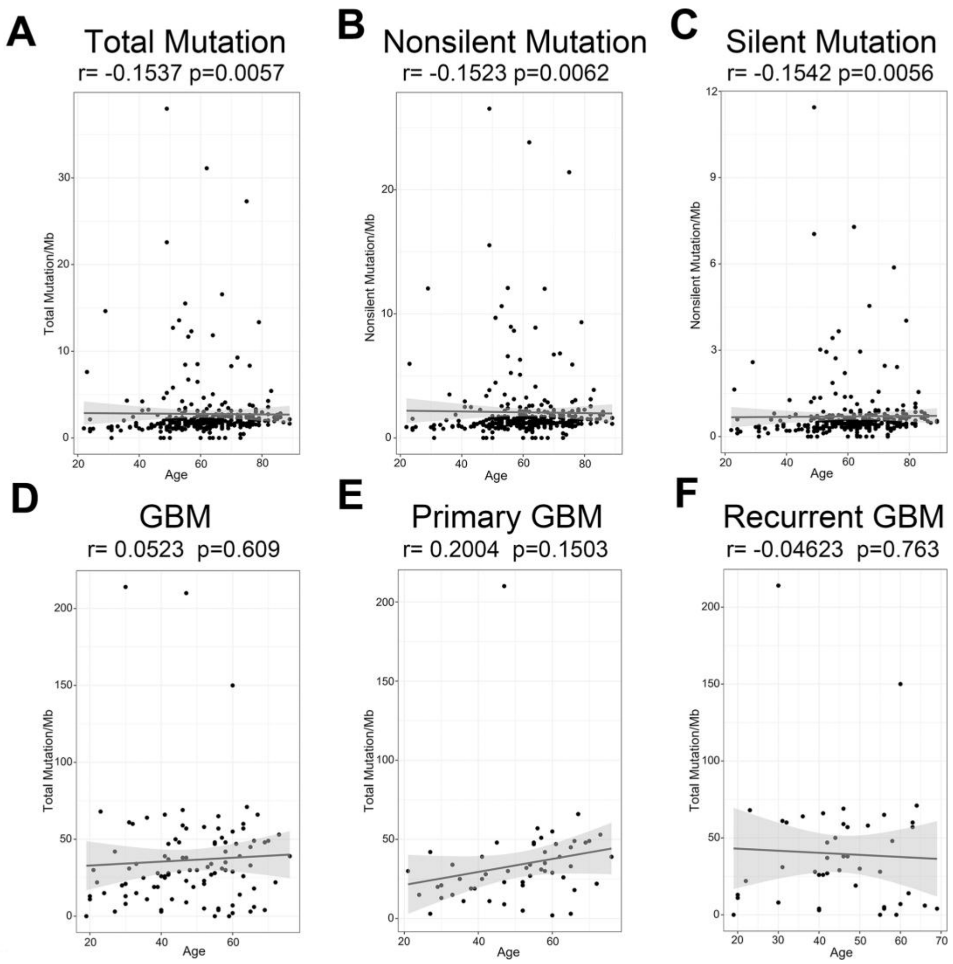

Supplementary Figure 1. Correlation analysis between age and gene mutation counts in primary GBM. (**A**–**C**) There was a negative correlation between age and counts of total mutation, non-silent mutation, or silent mutation in primary GBM in TCGA database. (**D**–**F**) There was no significant correlation between age and counts of total mutation in primary and recurrent GBM in CGGA database. The statistical significance was assessed by Pearson correlation analysis.

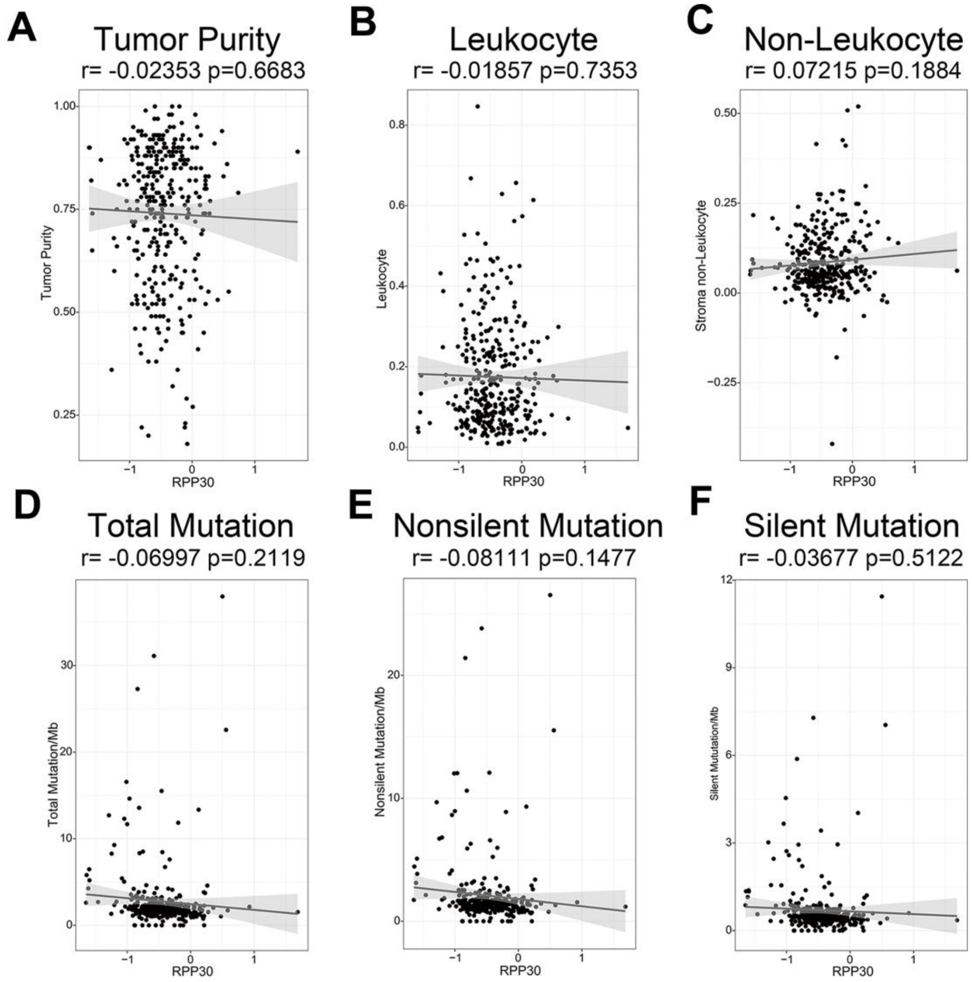

Supplementary Figure 2. RPP30 showed no correlation with tumor purity and mutation counts in primary GBM. (**A**–**C**) There was no significant correlation between RPP30 and tumor purity, leukocyte, or non-leukocyte ratios in primary GBM. (**D**–**F**) There was no significant correlation between RPP30 and counts of total mutation, non-silent mutation, or silent mutation in primary GBM. The statistical significance was assessed by Pearson correlation analysis.

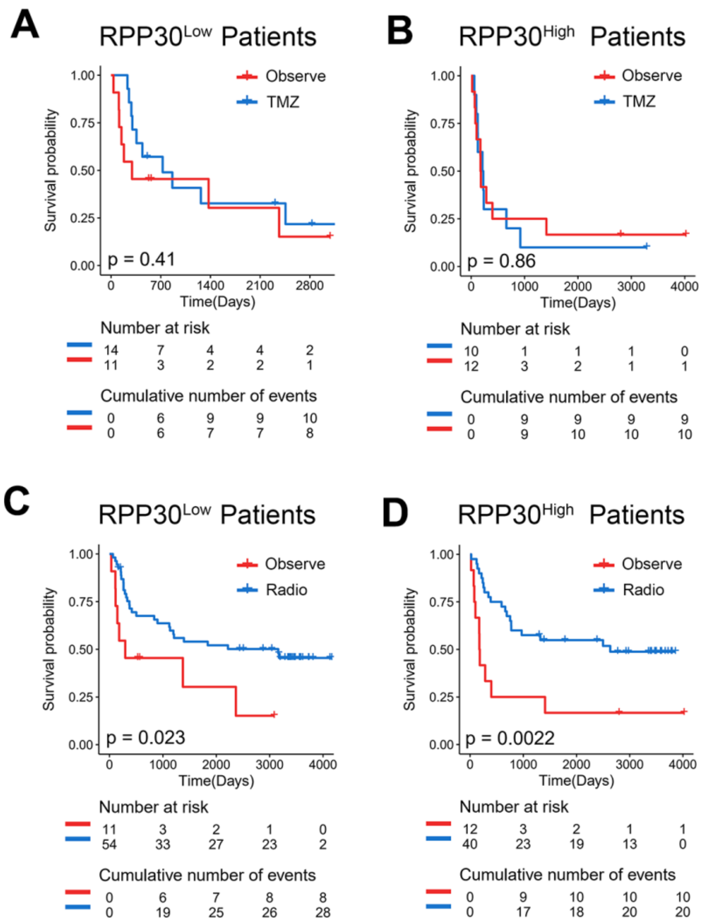

Supplementary Figure 3. The expression of RPP30 was independent of the sensitivity of postoperative radiotherapy and temozolomide in primary GBM. (**A**, **B**) Kaplan-Meier curves showed patients with high or low RPP30 expression cannot benefit from postoperative temozolomide alone. (**C**, **D**) Kaplan-Meier curves showed both patients with high or low RPP30 expression can benefit from postoperative radiotherapy alone.

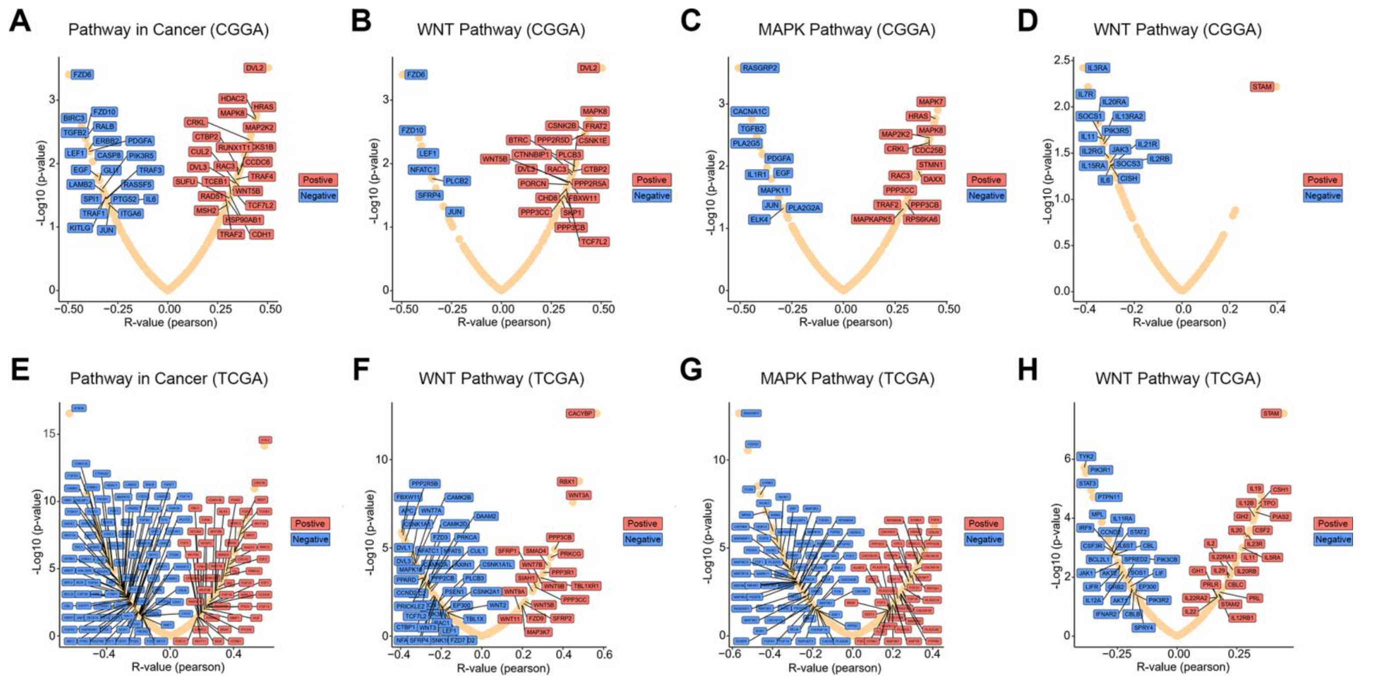

Supplementary Figure 4. The expression of RPP30 was significantly correlated with the expression of genes in cancer-related pathways. Genes in cancer-related pathways significantly correlated with the expression of RPP30 were displayed in CGGA database (**A**–**D**) and TCGA database (**E**–**H**). Genes that were significantly positive/negative correlated with the expression of RPP30 were marked in red/blue. Non-significantly correlated genes were not shown. The statistical significance was assessed by Pearson correlation analysis. p-value less than 0.05 was considered statistically significant.

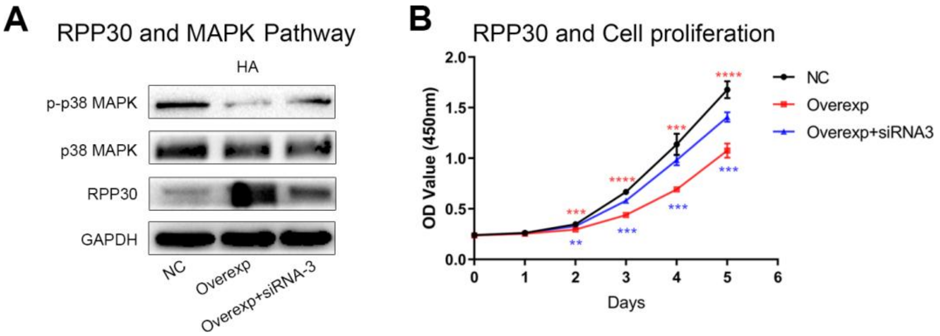

Supplementary Figure 5. RPP30 regulated activation of MAPK pathway and cell proliferation in vitro. (**A**) Western blot showed over-expression of RPP30 led to decreased expression of p-p38 in HA cells. The expression of p-p38 was restored by specific knockdown of RPP30 expression. (**B**) Cell proliferation ability significantly decreased by over-expression of RPP30 in HA cells (Overexp vs. NC, the statistical results were marked in red). The cell proliferation ability could be partially restored by specific knockdown of RPP30 expression (Overexp+siRNA3 vs. Overexp, the statistical results were marked in blue). **: $p<0.01$. ***: $p<0.001$. ****: $p<0.0001$.
