# Supplementary material for: RPP30, a transcriptional regulator, is a potential pathogenic factor in glioblastoma
Source: Aging (Albany NY). 2020 Jul 23;12(16):16155–71. doi: 10.18632/aging.103596 (PMC7485703; doi:10.18632/aging.103596)
Supplement: Supplementary Tables 2 and 3 [file aging-12-103596-s002..pdf]

SUPPLEMENTARY TABLES

Supplementary Table 2. Primer sequence of RPP30.

	Sequence (5' -> 3')
Forward Primer	ACCTTGGCTATTCAGTTGTTGC
Reverse Primer	TGCTCTCAAAACATTGCAGTGA

Supplementary Table 3. Correlation analysis between RPP30 and transcription-related genes in CGGA and TCGA databases.

GO Terms		CGGA database		TCGA database	
	Genes	r	P (two-tailed)	r	P (two-tailed)
GO:0000184~nuclear-transcribed mRNA catabolic process, nonsense-mediated decay	EIF3E	0.4381	<0.0001	0.3048	<0.0001
	RPL18	0.3814	<0.0001	0.2466	<0.0001
	RPL22	0.3788	<0.0001	0.2248	<0.0001
	RPL26	0.421	<0.0001	0.3184	<0.0001
	RPL36A	0.435	<0.0001	0.3424	<0.0001
	RPL37	0.3646	<0.0001	0.2828	<0.0001
	RPL5	0.4503	<0.0001	0.5465	<0.0001
	RPL6	0.4435	<0.0001	0.2098	<0.0001
	RPL7A	0.427	<0.0001	0.394	<0.0001
	RPS15A	0.4353	<0.0001	0.3544	<0.0001
	RPS24	0.4611	<0.0001	0.5601	<0.0001
GO:0002181~cytoplasmic translation	RPL22	0.3788	<0.0001	0.2248	<0.0001
	RPL26	0.421	<0.0001	0.3184	<0.0001
	RPL36A	0.435	<0.0001	0.3424	<0.0001
	RPL6	0.4435	<0.0001	0.2098	<0.0001
GO:0006364~rRNA processing	DDX21	0.5104	<0.0001	0.2219	<0.0001
	EXOSC1	0.4517	<0.0001	0.6202	<0.0001
	EXOSC2	0.3932	<0.0001	0.3335	<0.0001
	RPL18	0.3814	<0.0001	0.2466	<0.0001
	RPL22	0.3788	<0.0001	0.2248	<0.0001
	RPL26	0.421	<0.0001	0.3184	<0.0001
	RPL36A	0.435	<0.0001	0.3424	<0.0001
	RPL37	0.3646	<0.0001	0.2828	<0.0001
	RPL5	0.4503	<0.0001	0.5465	<0.0001
	RPL6	0.4435	<0.0001	0.2098	<0.0001
	RPL7A	0.427	<0.0001	0.394	<0.0001
	RPS15A	0.4353	<0.0001	0.3544	<0.0001
	RPS24	0.4611	<0.0001	0.5601	<0.0001
	SIRT1	0.4413	<0.0001	0.3178	<0.0001
	WDR12	0.3704	<0.0001	0.2962	<0.0001
GO:0006412~translation	EIF4EBP2	0.464	<0.0001	0.3343	<0.0001
	MRPL1	0.3958	<0.0001	0.4443	<0.0001
	MRPL43	0.5936	<0.0001	0.5809	<0.0001

	MRPS16	0.4003	<0.0001	0.5071	<0.0001
	RPL10L	0.3801	<0.0001	0.424	<0.0001
	RPL18	0.3814	<0.0001	0.2466	<0.0001
	RPL22	0.3788	<0.0001	0.2248	<0.0001
	RPL26	0.421	<0.0001	0.3184	<0.0001
	RPL36A	0.435	<0.0001	0.3424	<0.0001
	RPL37	0.3646	<0.0001	0.2828	<0.0001
	RPL5	0.4503	<0.0001	0.5465	<0.0001
	RPL6	0.4435	<0.0001	0.2098	<0.0001
	RPL7A	0.427	<0.0001	0.394	<0.0001
	RPS15A	0.4353	<0.0001	0.3544	<0.0001
	RPS24	0.4611	<0.0001	0.5601	<0.0001
	ZNF525	0.3792	<0.0001	0.275	<0.0001
GO:0006413~translational initiation	EIF3E	0.4381	<0.0001	0.3048	<0.0001
	EIF3H	0.448	<0.0001	0.3047	<0.0001
	PAIP1	0.4281	<0.0001	0.4109	<0.0001
	RPL18	0.3814	<0.0001	0.2466	<0.0001
	RPL22	0.3788	<0.0001	0.2248	<0.0001
	RPL26	0.421	<0.0001	0.3184	<0.0001
	RPL36A	0.435	<0.0001	0.3424	<0.0001
	RPL37	0.3646	<0.0001	0.2828	<0.0001
	RPL5	0.4503	<0.0001	0.5465	<0.0001
	RPL6	0.4435	<0.0001	0.2098	<0.0001
	RPL7A	0.427	<0.0001	0.394	<0.0001
	RPS15A	0.4353	<0.0001	0.3544	<0.0001
	RPS24	0.4611	<0.0001	0.5601	<0.0001
GO:0006614~SRP-dependent cotranslational protein targeting to membrane	RPL18	0.3814	<0.0001	0.2466	<0.0001
	RPL22	0.3788	<0.0001	0.2248	<0.0001
	RPL26	0.421	<0.0001	0.3184	<0.0001
	RPL36A	0.435	<0.0001	0.3424	<0.0001
	RPL37	0.3646	<0.0001	0.2828	<0.0001
	RPL5	0.4503	<0.0001	0.5465	<0.0001
	RPL6	0.4435	<0.0001	0.2098	<0.0001
	RPL7A	0.427	<0.0001	0.394	<0.0001
	RPS15A	0.4353	<0.0001	0.3544	<0.0001
	RPS24	0.4611	<0.0001	0.5601	<0.0001
